# Address—Introductory to the Course of Lectures at the Ohio Dental College, Oct. 15, 1873

**Published:** 1873-11

**Authors:** J. S. Cassidy

**Affiliations:** Professor of Chemistry


### DENTAL REGISTER.

Vol. XXVII.] NOVEMBER, 1873. [No. 11.

### ADDRESS—INTRODUCTORY TO THE COURSE OF LECTURES AT THE OHIO DENTAL COLLEGE, OCT. 15, 1873.

BY J. S. CASSIDY, PROFESSOR OF CHEMISTRY.

Gentlemen :—Permit me to assure you, that it is a pleasure to me, as well as a duty, to act on this occasion as the representative of my colleagues of the faculty, and extend to you a cordial welcome.

The beginning of a course of lectures in an institution of this kind is an event of considerable interest to all parties concerned. Especially is it so to the student, who, for the first time, enters upon a regular course of training, preparatory to commencing the legitimate practice of his chosen vocation. His position is entirely new in its relations to his past experience. He comes a stranger and finds himself in the midst of confidential friends. The unknown faces that surround him to-day, and which he scans with the interested intensity of the practised physiognomist, will greet him to-morrow, with the smile of familiar recognition due to an old acquaintance. There is unrestrained interchange of newly acquired ideas, and to the novice in the ways that are difficult to understand, assistance and encouragement are most lavishly extended, so that, indeed, unless his mind be prematurely soured by the "acid secretion" of a misanthropic brain, he will find among his grown-up, incipiently aged classmates, a sufficiency of the joyous element of human nature to render the time of his association with them one of the pleasantest periods of his existence.

But while it is eminently proper that you should seek every opportunity for the cultivation of enjoyment, and of the social freedom that always exists among those who are daily brought together in the pursuit of the same laudable object, and whose spirits possess the redundant buoyancy of early manhood, it is well to remember that you have not come here to enjoy a holiday. You begin to-day the systematic study of science and art, in their application to the requirements of dentistry, and the field to be traveled over in this connexion is so large, so rich in essentially useful facts, that he who would garner the sheaves of knowledge must be no laggard; he must work earnestly and incessantly. For in order to merit the high standing to which every student should desire to attain, there must be on his part close application, untiring industry and diligence; not, however, to the extent of weariness for that would tend to render negative the results you wish to accomplish. "All work and no play makes Jack a dull boy."

Permanent success in any calling is attained only by means of well-directed, persistent labor. The fortuitous circumstances by which apparently many individuals are elevated above their peers in the social and political world, cannot be ascribed as either the remote or proximate cause of the success which invariably awaits the devoted student. Within his own brain must be arranged and analyzed whatever is perplexing or formidable in the technicalities or nomenclature of descriptive science, and their uses in practical art. And herein is his reward immediate and lasting. His labor becomes "a labor of love," and his newly acquired knowledge an ever present source of satisfaction, gratifying to his pride, ennobling and enriching his mind and heart with an exalted pleasure, sweet, and unalloyed, and unceasing.

You will find as we proceed in the session, that the course of instruction you are called upon to follow has been arranged with a view of your complete and entire mastery of all the subjects presented, and hence to preparing you in the most thorough manner for commencing your special practice. Your curriculum has been selected with careful reference to obtaining the best practical results. There is no time for you to spare to deal in abstruse ideas. However much the world of science may be indebted to the transcendental dreamers of the past and present, the world wrapped in its utilitarian garb refuses to acknowledge the benefits it has derived from their labors, except as it appropriates to itself their victories over the unknown. You are not to wing your fancies into regions unexplored by others. There is so much already known of the useful and the beautiful within the limits of investigated truth, that for you to go outside its bounds and into the domains of the hypothetical would be a waste of precious time. It is ,of course, the high prerogative of every individual to enter and investigate the illimitable fields of science and art and literature, according to his capacity and pleasure, but the object of your presence here is not of a general nature ; it is to obtain, if possible, all the facts now known relating to the theory and practice of dentistry. You are to lay a deep and broad and solid foundation upon which you can stand as capable and honest men ; and upon which you can base your future studies and investigation, so as not only to insure a profitable and speedy return for your labor, but also to acquire a systematic method of obtaining an easy and pleasant acquaintanceship with truth.

Your time is limited to a certain specified period, so also must your studies be limited and definite as to quantity and quality. It must not, however, be understood that your attention will be confined to the consideration of subjects that possess direct and exclusive bearing upon the truth. They, indeed, are the central axis around which must revolve your thoughts and to their study you must apply all the knowledge and ability you can acquire. You are taught to study their development from the moment the microscope is able to detect their infinitesimal beginning as parts of embryonic life on to the period of their full maturity ; to acquaint yourselves with the causes and progress of the diseases to which they are subjected, and the surest means at our command to be employed in preventing their destruction. But primarily a knowledge of some of the collateral sciences is essentially needed. You must, for instance, become familiar with general anatomy. The intimate relations existing between the different parts of the body —all alike subject to the vicissitudes of disease, and finally to the certainty of death—compel a careful examination of all in order to act intelligently in the treatment of a part. The specialist cannot restrict himself to the exclusive study of his favorite organ : he cannot separate it from its connexion with surrounding parts, and, thus isolated, give an intelligent explanation of the cause and progress, and probable termination of any morbid condition with which it may be afflicted. He must first acquaint himself with the complete mechanical arrangement of the body in all its grand and beautiful entirety, and then his more intimate study of special portions becomes a work of easy and clear conception, fully appreciated, because understandingly followed. Side by side with anatomy is placed the important science of physiology ; important and of deep interest to all, because it treats of the natural functions performed by the living organism, by which life is propagated and maintained. It is a science which seeks satisfactory explanation of the observed phenomena occurring in all living things. It illuminates and discloses to your delighted view some of the recesses of hitherto darkened chambers wherein you see and comprehend the normal processes of natural action continuously at work ; where every part is attuned to play in unison with the universal harmony of nature.

There is no branch of science that merits more of man's studious consideration than physiology ; nor can he devote himself to a more pleasing task than to studying its teachings; but aside from the exquisite pleasure you will derive in pursuing your studies on this subject, it is of the utmost importance that you should become familiar with the laws of health, with the natural functions performed by the different organs. 'Tis only by your previous knowledge of normal action in possibly remote parts, that can enable you hereafter in many cases to detect the difference between a nervous sympathy and an actual disease of the organs entrusted to your care.

The mere administration of an anæsthetic for the purpose of procuring the unnatural condition of insensibility to pain during the most trivial operation in surgery, requires, on the part of the operator, a higher degree of proficiency in knowledge of the laws of physiological action—and hence the ability to discriminate correctly in every instance, that the life of the patient may be undoubtedly assured—than practitioners of long and eminent standing pretend to possess. Witness the numerous sad cases of death by chloroform, notwithstanding every precaution, and where *post mortem* examination utterly fails to detect the slightest lesion of any of those organs wherein death is supposed to commence.

I allude to this subject not to call in question the high attainments of any one, but for the purpose of impressing upon you the necessity of applying yourselves while here with all the diligence you are capable of to the careful study of man's strange mechanism and the laws that govern it.

To aim only at the high standard demanded by the institution which you hope ere long to claim as your Alma Mater, or in plain language to get through just by "the skin of your teeth," is unworthy the aspirations of honest ambition. Who are they whose unfitness to pursue their professed vocation is recorded in heaven and perhaps, also, in the world of perdition? Some of them, alas! are graduates of medicine and dentistry, whose "diploma in office will show." Men whose better instincts—if they ever had any—have been perverted; and whose only recognized ability is the power of deceiving a confiding public. Men whose daily existence is a lie and a fraud, a travesty on honesty and common sense. You have a higher and holier motive to impel you to greater exertion than the simple expectation of obtaining the privileges that a diploma confers.

There is another branch of study which will demand your most earnest attention, and of which we will have a good deal to say during the next few months—I allude to chemistry. Born in the laboratories of the dreaming alchemists, and nurtured by their persistent efforts to succeed in discovering the mythical elixir of life, and in transmuting certain metals into g ld, it has grown steadily apace with modern progress, and now its immense development in every country on the globe has added immeasurably to the comfort and happiness of mankind.

Anatomy, physiology and chemistry are the foundation stones upon which is reared the noble edifice of medicine and surgery. It is by means of your acquaintance with these three sciences, that you will be enabled to pursue with profit your studies in pathology and therapeutics, and indeed in all the other branches of study that will demand your attention while here.

I have mentioned only a portion of the studies that, from their nature, are most calculated to worry the dental student. His mind is far more at home in learning facts pertaining directly to the nature and preservation of the natural teeth, and to the different modes of constructing artificial ones to supply Nature's loss. Right here, it seems to me, comes in the principal secret of success which has marked the career of nearly all who have availed themselves of the advantages offered for instruction in the necessary collateral sciences, and in the details of practical art, as taught in properly conducted dental colleges. The union of theoretical and practical teaching, the latter understood as including the clinical and mechanical departments, keeping up, so to speak, an equilibrium between mental and manual labor, the one serving as an antidote to the wearisome exclusiveness of the other, could not be otherwise than conducive to the very best interests of the student. The lessons he receives in theory are immediately applied in actual practice, and thus he is conducted along, step by step, from the first dim passage of doubt and uncertainty into the bright atmosphere of hope, and on to the certainty of final triumph. That is, provided always he possesses the requisite natural ability.

It requires no more than ordinary common sense, united to more or less determination, to succeed in obtaining the mastery over the most difficult studies; but there is also something else needed, in order to become a good dentist. It is what is vulgarly termed mechanical skill. In what attribute is the surgeon superior to the general physician? Certainly not in superior knowledge of structure, for the ordinary physician is also well informed in anatomy; nor is it necessarily in long experience, for many a young doctor is far more expert with the scalpel than is his preceptor; neither does it consist in the power of holding in abeyance the tenderer feelings of humanity, for as a rule the most skillful surgeon is the most sympathetic and the kindest of men. My answer is, the attribute consists simply in his possessing intuitive mechanical skill; and so also to the dentist, this peculiar faculty is an imperative necessity. Without it, the so-called dentist is a bungling nuisance; and with it to obey the commands of the knowledge acquired by scientific education, your future success will be well assured.

It is, therefore, your duty, gentlemen, to cultivate and train this gift to the highest degree of artistic excellence, to be ardent in seeking close familiarity with all the truths which science teaches, and my word for it, you will go forth from these halls as men worthy the highest standing in the brotherhood of dentists. You are here to qualify yourselves for the practice of a profession that has received contribution from nearly every mechanical pursuit; it has searched through the inexhaustible stores of its parent medicine, holding fast to that which is good, and in return, it has given to the world some of the most useful discoveries, so potent in assisting Nature in her continual conflict with disease. And notwithstanding that it has thus been productive of magnificent results, and has been so extended that the labor of years is required to enable the mind of the student to grasp it in its various branches, its capabilities for the future are probably greater than its development in the past, and there are few departments in which man's desire to investigate can find freer play, or his labor more certainty of ultimate reward.

